# Mobile Contactless Fingerprint Recognition: Implementation, Performance and Usability Aspects

**DOI:** 10.3390/s22030792

**Published:** 2022-01-20

**Authors:** Jannis Priesnitz, Rolf Huesmann, Christian Rathgeb, Nicolas Buchmann, Christoph Busch

**Affiliations:** 1 da/sec—Biometrics and Internet Security Research Group, Hochschule Darmstadt, Schöfferstraße 8b, 64295 Darmstadt, Germany; christian.rathgeb@h-da.de (C.R.); christoph.busch@h-da.de (C.B.); 2 UCS—User-Centered Security Research Group, Hochschule Darmstadt, Schöfferstraße 8b, 64295 Darmstadt, Germany; rolf.huesmann@h-da.de; 3 Fraunhofer AISEC, Breite Straße 12, 14199 Berlin, Germany; nicolas.buchmann@aisec.fraunhofer.de

**Keywords:** biometrics, fingerprint recognition, contactless fingerprint, usability, biometric performance

## Abstract

This work presents an automated contactless fingerprint recognition system for smartphones. We provide a comprehensive description of the entire recognition pipeline and discuss important requirements for a fully automated capturing system. In addition, our implementation is made publicly available for research purposes. During a database acquisition, a total number of 1360 contactless and contact-based samples of 29 subjects are captured in two different environmental situations. Experiments on the acquired database show a comparable performance of our contactless scheme and the contact-based baseline scheme under constrained environmental influences. A comparative usability study on both capturing device types indicates that the majority of subjects prefer the contactless capturing method. Based on our experimental results, we analyze the impact of the current COVID-19 pandemic on fingerprint recognition systems. Finally, implementation aspects of contactless fingerprint recognition are summarized.

## 1. Introduction

Fingerprints are one of the most important biometric characteristic due to their known uniqueness and persistence properties. Fingerprint recognition systems are not only used worldwide by law enforcement and forensic agencies, they are also deployed in mobile devices as well as in nationwide applications. The vast majority of fingerprint capturing schemes requires contact between the finger and the capturing device’s surface. These systems suffer from distinct problems, e.g., low contrast caused by dirt or humidity on the capturing device plate or latent fingerprints of previous users (ghost fingerprints). Especially in multi-user applications, hygienic concerns lower the acceptability of contact-based fingerprint systems and hence limit their deployment. In a comprehensive study, Okereafor et al. [1] analyzed the risk of an infection by contact-based fingerprint recognition schemes and the hygienic concerns of their users. The authors concluded that contact-based fingerprint recognition carries a high risk of an infection if a previous user has contaminated the capturing device surface, e.g., with the SARS-CoV-2 virus.

To tackle these shortcomings of contact-based schemes, contactless fingerprint recognition systems have been researched for more than a decade. Contactless capturing schemes operate without any contact between the finger and the capturing device. Several contributions to the research area have paved the way for a practical implementation of contactless capturing schemes. Specialized stationary capturing devices based on multi-camera setups combined with powerful processing have already been implemented in a practical way [2]. However, to the best of the authors’ knowledge, no approach to a comprehensive usability-oriented mobile contactless fingerprint recognition scheme based on off-the-shelf components such as smartphones has been documented so far.

In this work, we propose a mobile contactless fingerprint recognition scheme for smartphones. Our contributions can be summarized as follows:

- We present the first fully automated four-finger capturing and preprocessing scheme with integrated quality assessment in form of an Android app. A description of every implementation step of the preprocessing pipeline is given.
- To benchmark our proposed system, we acquired a database under real-life conditions. A number of 29 subjects was captured by two contactless capturing devices in different environmental situations. Contact-based samples were also acquired as baseline.
- We further evaluate the biometric performance of our acquired database and measure the interoperability between both capturing device types.
- We provide a first comparative study about the usability of contactless and contact-based fingerprint recognition schemes. The study was conducted after the capture sessions and reports the users’ experiences in terms of hygiene and convenience.
- Based on our experimental results, we elaborate on the impact of the current COVID-19 pandemic on fingerprint recognition in terms of biometric performance and user acceptance. Furthermore, we summarize implementation aspects which we consider as beneficial for mobile contactless fingerprint recognition.

The whole capturing, processing, and recognition pipeline discussed in this work is made publicly available for research purposes^1^. Moreover, interested researchers are welcome to hand-in and benchmark their algorithms on our acquired database^2^.

The rest of the paper is structured as follows: Section 2 gives an overview of contactless end-to-end schemes proposed in the scientific literature. In Section 3, the proposed processing pipeline is presented. In Section 4, we describe our experimental setup and provide details about the captured database and the usability study. The results of our experiments are reported in Section 5. The influence of the COVID-19 pandemic on fingerprint recognition is discussed in Section 6. Section 7 discusses implementation aspects. Finally, Section 8 concludes.

## 2. Related Work

In this section, we present an overview of contactless fingerprint recognition workflows. Here, we focus on end-to-end solutions which present a whole recognition pipeline from capturing to comparison. Table 1 summarizes the most relevant related works and their implementation aspects. As Table 1 indicates, the proposed methods are very different in terms of capturing device, fingerprint processing, recognition pipeline, and user convenience. In addition, the acquired databases vary in terms of size, illumination, and environmental influences. For this reason, a fair comparison of the biometric performance reported in the listed works is misleading and is therefore avoided.

The research on contactless fingerprint recognition has evolved from bulky single-finger devices to more convenient multi-finger capturing schemes. The first end-to-end approaches with prototypical hardware setups were presented by Hiew et al. [3] and Wang et al. [4]. Both works employed huge capturing devices for one single-finger acquisition within a hole-like guidance. A more recent approach by Attrish et al. [5] also used a box-like capturing setup and proposed processing which is implemented in an embedded hardware unit.

**Table 1:** Overview of selected recognition workflows with implementation aspects. (Device type: P = prototypical hardware, S = smartphone, W = webcam).

Authors	Year	Device Type	Mobile/ Stationary	Multi-Finger Capturing	Automatic Capturing	Free Finger Positioning	Quality Assessment	On-Device Processing	Usability Evaluation
Hiew et al. [3]	2007	P	S	N	N	N	N	N	N
Piuri and Scotti [6]	2008	W	S	N	N	N	N	N	N
Wang et al. [4]	2009	P	S	N	N	N	N	N	N
Kumar and Zhou [7]	2011	W	S	N	N	N	N	N	N
Noh et al. [8]	2011	P	S	Y	Y	N	N	N	Y
Derawi et al. [9]	2012	S	S	N	N	N	N	N	N
Stein et al. [10]	2013	S	M	N	Y	Y	N	Y	N
Raghavendra et al. [11]	2014	P	S	N	N	Y	N	N	N
Tiwari and Gupta [12]	2015	S	M	N	N	Y	N	N	N
Sankaran et al. [13]	2015	S	M	N	Y	N	N	N	N
Carney et al. [14]	2017	S	M	Y	Y	N	N	Y	N
Deb et al. [15]	2018	S	M	N	Y	Y	Y	Y	N
Weissenfeld et al. [16]	2018	P	M	Y	Y	Y	N	Y	Y
Birajadar et al. [17]	2019	S	M	N	Y	N	N	N	N
Attrish et al. [5]	2021	P	S	N	N	N	N	Y	N
Kauba et al. [18]	2021	S	M	Y	Y	Y	N	Y	N
Our method	2021	S	M	Y	Y	Y	Y	Y	Y

For remote user authentication, Piuri et al. [6] and Kumar et al. [7] investigated the use of webcams as fingerprint-capturing device. Both schemes showed a very low EER in experimental results. However, the database capturing process was not reported precisely. In addition, the usability and user acceptance of such an approach should be further investigated.

More recent works use smartphones for contactless fingerprint capturing. Here, a finger image is taken by a photo app and is manually transferred to a remote device where the processing is performed [12,13]. The improvement of the camera and processing power in current smartphones has made it possible to capture multiple fingers in a single capture attempt and process them on the device. Stein et al. [10] showed that it is feasible for the automated capturing of a single finger image using a smartphone. Carney et al. [14] presented the first four-finger capturing scheme. Weissenfeld et al. [16] proposed a system with a free positioning of four fingers in a mobile prototypical hardware setup. In a later work, Kauba et al. [18] showed that the recognition workflow also works on a smartphone.

In summary, Table 1 indicates that the evolution of contactless fingerprint technologies has moved towards mobile out-of-the-box devices. It can also be observed that a more convenient and practically relevant recognition process is increasingly becoming the focus of research. For a comprehensive overview on the topic of contactless fingerprint recognition, including publications which consider only parts of the recognition pipeline, the reader is referred to [19,20].

## 3. Mobile Contactless Recognition Pipeline

**Figure 1:**
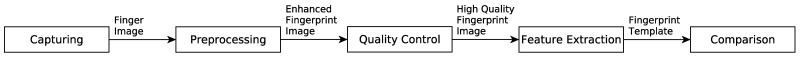
Overview of the most relevant steps of our proposed method.

An unconstrained and automated contactless fingerprint recognition system usually requires a more elaborated processing compared to contact-based schemes. Figure 1 gives an overview of the key processing steps of the proposed recognition pipeline. Our method features on-device capturing, preprocessing, and quality assessment, whereas the biometric identification workflow is implemented on a back-end system. This section describes each component of the recognition pipeline in detail. The proposed method combines four implementation aspects seen as beneficial for an efficient and convenient recognition:

- An Android application running on a smartphone which continuously captures finger images as candidates for the final fingerprints and provides user feedback.
- A free positioning of the four inner-hand fingers without guidelines or a framing.
- An integrated quality assessment which selects the best-suited finger image from the list of candidates.
- A fully automated processing pipeline which processes the selected candidate to fingerprints ready for the recognition workflow.

### 3.1. Capturing

The vast majority of mobile contactless recognition schemes rely on state-of-the-art smartphones as capturing devices. Smartphones offer a high-resolution camera unit, a powerful processor, and an integrated user feedback via display and speaker, as well as a mobile internet connection for on-demand comparison against centrally stored databases.

In our case, the capturing, as well as the processing, is embedded in an Android app. Once the recognition process is started, the application analyzes the live-view image and automatically captures a finger image if the quality parameters fit the requirements. The application is designed to automatically capture and process up to six images per second. The capturing module resizes the captured image to a fixed size of 1.920 × 1.080 pixels. This makes the processing pipeline more robust against the native resolution of the camera sensor and ensures that the capturing device is able to process the input images with a moderate system load. During capturing, the user is able to see his/her fingers through a live-view on the screen and is able to adjust the finger position. In addition, the capturing progress is displayed.

### 3.2. Segmentation of the Hand Area

Proposed strategies for the segmentation mainly rely on color and contrast. Many works use color models for segmenting the hand color from the background. Here, an Otsu’s adaptive threshold is preferable over static thresholding. Combinations of different color channels also show superior results compared to schemes based on one channel [21,22,23,24].

**Figure 2:**
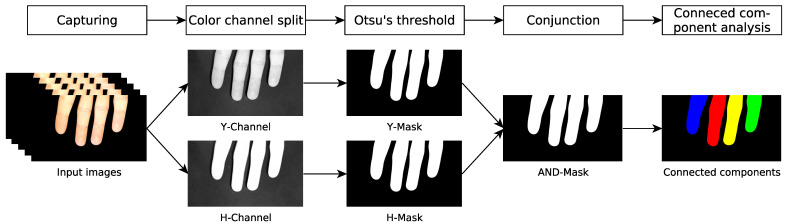
Overview of the segmentation of connected components from a continuous stream of input images.

Figure 2 presents an overview of the segmentation workflow. We adopt this method and analyze the Cr component of the yCbCr color model and the H component of the HSV color model. As a first step, we normalize the color channels to the full range of the histogram. Subsequently, the Otsu’s threshold determines the local minimum in the histogram curve. A binary mask is created where all pixel values below the threshold are set to black and all pixels above the threshold are set to white.

Additionally, our algorithm analyzes the largest connected components within the segmentation mask. Ideally, the segmentation mask should only contain one to four dominant components: from one hand area up to four finger areas, respectively. Our method also implements a plausibility check of the size, shape, and position of segmented areas.

### 3.3. Rotation Correction, Fingertip Detection, and Normalization

The rotation correction transforms every finger image in a way such that the final fingerprint image is oriented in an upright position.

**Figure 3:**
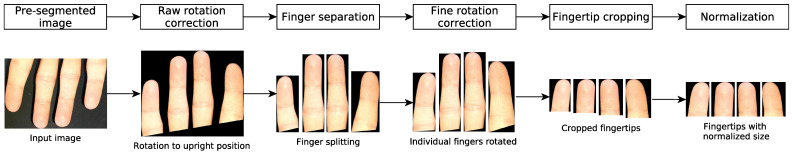
Overview of the coarse rotation correction, separation of fingerprint images from each other, fine rotation correction, fingertip cropping, and normalization of the fingerprint size.

**Figure 4:**
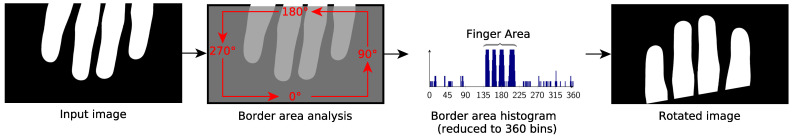
Detailed workflow of the coarse rotation correction.

Figure 3 presents an overview of the rotation correction, fingertip detection, and normalization. Our method features two rotation steps: First, a coarse rotation on the full hand, and second, a fine rotation on the separated finger. A robust separation and identification of the fingers requires that the hand is rotated to an upright position. Here, the image border of the binary segmentation mask is analyzed. Many white border pixels indicate that the hand is placed into the sensor area from this particular direction. For this reason, we search for the border area with the most white pixels and calculate a rotation angle from this coordinate. Figure 4 illustrates this method.

After the coarse rotation, the fingertips are separated. To this end, the number of contours of considerable size is compared to a preconfigured value. If there are fewer contours than expected, it is most likely that the finger images contain part of the palm of the hand. In this case, pixels are cut out from the bottom of the image and the sample is tested again. In the case of more considerable contours, the finger image is discarded in order to avoid processing wrong finger-IDs.

An upright rotated hand area does not necessarily mean that the fingers are accurately rotated, because fingers can be spread. A fine rotation is computed on every finger image to correct such cases. Here, a rotated minimal rectangle is placed around every dominant contour. This minimal rectangle is then rotated into an upright position.

Additionally, the height of each finger image needs to be reduced to the area which contains the fingerprint impression. Other works have proposed algorithms which search for the first finger knuckle [25,26]. We implemented a simpler method which cuts the height of the finger image to the double of its width. In our use case, this method leads to slightly less accurate result but is much more robust against outliers.

Contactless fingerprint images captured in different sessions or processed by different workflows do not necessarily have the same size. The distance between sensor and finger defines the scale of the resulting image. For a minutiae-based comparison, it is crucial that both samples have the same size. Moreover, in a contactless-to-contact-based interoperability scenario, the sample size has to be aligned to the standardized resolution, e.g., 500 dpi. Therefore, we normalize the fingerprint image to a width of 300 pixels. This size refers to a ridge-to-ridge distance of approximately seven pixels, which corresponds to the distance of contact-based fingerprints captured with 500 dpi.

Together with the information regarding which hand is captured, an accurate rotation correction also enables a robust identification of the finger-ID, e.g., index, middle, ring, or little finger. Assuming that the capture subject holds the capturing device in an upright position, we analyze whether a left or right hand is presented. Subsequently, our algorithm automatically labels the fingerprint images with the corresponding finger-ID.

### 3.4. Fingerprint Processing

**Figure 5:**
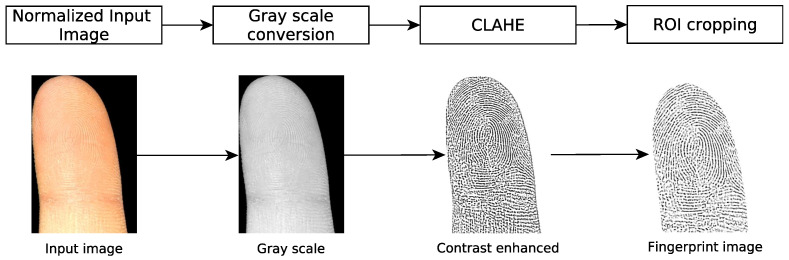
Overview of grayscale conversion, application of CLAHE, and cropping of the fingerprint region Of interest (ROI). This process is executed on every separated finger.

The preprocessed fingerprint image is aligned to resemble the impression of a contact-based fingerprint. Figure 5 presents the conversion from a finger image to a contactless fingerprint. We use the Contrast Limited Adaptive Histogram Equalization (CLAHE) on a grayscale-converted fingerprint image to emphasize the ridge-line characteristics.

Preliminary experiments showed that the used feature extractor detects many false minutiae at the border region of contactless fingerprint samples. For this reason, we crop approximately 15 pixels of the border region; that is, the segmentation mask is dilated in order to reduce the size of the fingerprint image.

### 3.5. Quality Assessment

Quality assessment is a crucial task for contact-based and contactless fingerprint recognition schemes. We distinguish between two types of quality assessment: An integrated plausibility check at certain points of the processing pipeline and a quality assessment on the final sample.

The integrated plausibility check is an essential precondition for a successful completion of an automated recognition scheme. It ensures that only samples which passed the check of a processing stage are handed over to the next stage. In the proposed preprocessing pipeline, we implement three plausibility checks:

- Segmentation: Analysis of the dominant components in the binary mask. Here, the amount of dominant contours, as well as their shape, size, and position are analyzed. In addition, the relative positions to each other are inspected.
- Capturing: Evaluation of the fingerprint sharpness. A Sobel filter evaluates the sharpness of the processed grayscale fingerprint image. A square of 32 × 32 pixels at the center of the image is considered. A histogram analysis then assesses the sharpness of the image.
- Rotation, cropping: Assessment of the fingerprint size. The size of the fingerprint image after the cropping stage shows whether the fingerprint image is of sufficient quality.

The combination of these plausibility checks has shown to be robust and accurate in our processing pipeline. Every sample passing all three checks is considered as a candidate for the final sample. For every finger-ID, five samples are captured and processed. All five samples are finally assessed by NFIQ2.0 [27] and the sample with the highest-quality score is considered as the final sample. An assessment on the applicability of NFIQ2.0 on contactless fingerprint samples is presented in [28].

### 3.6. Feature Extraction and Comparison

As mentioned earlier, the presented contactless fingerprint processing pipeline is designed in a way that obtained fingerprints are compatible with existing contact-based minutiae extractors and comparators. This enables the application of existing feature extraction and comparator modules within the proposed pipeline and facilitates a contact-based-to-contactless fingerprint comparison. Details of the employed feature extractor and comparator are provided in Section 5.

## 4. Experimental Setup

To benchmark our implemented app, we conducted a data acquisition along with a usability study. Each volunteering subject first participated in a data acquisition session and then was asked to answer a questionnaire.

### 4.1. Database Acquisition

We acquired a database to evaluate our proposed recognition pipeline under real-life conditions. The database capturing was carried out during the COVID-19 pandemic. For this reason, the acquisition setup had to meet institutional regulations, e.g., the capture subjects had to handle the capturing devices without close interaction of the instructor. This simulated a semisupervised capturing process and fulfilled the hygienic regulations during the database acquisition. It should be mentioned that the recruiting of participants was challenging due to general hygienic concerns. Therefore, the captured database is rather small compared to others, e.g., of Lin and Kumar [29].

**Figure 6:**
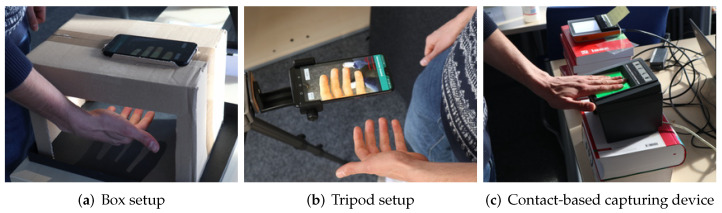
Capturing device setups during our experiments.

For the capturing of contactless samples, two different setups were used: Firstly, a box-setup simulates a predictable dark environment. Nevertheless, the subject was still able to place their fingers freely, c.f. Figure 6a. Secondly, a tripod setup simulates a fully free capturing setup where the instructor or the subject holds the capturing device, c.f. Figure 6b.

For the contactless database capturing, we used two different smartphones: the Huawei P20 Pro (tripod setup) and the Google Pixel 4 (box setup). The finger images are captured with the highest possible resolution and downscaled as described in Section 3.1. Our proposed application is designed to run on most state-of-the-art Android devices. The downscaling of the input images reduces the influence of different capturing device resolutions and ensures that the system load is at a moderate level. For this reason, the influence of the used smartphones on our experimental results are considered as minor. An overview of the technical specifications of the used contactless capturing devices is shown in Table 2. Both devices captured and processed six frames per second, which resulted in an average system load of less than 85% on both systems.

In addition, contact-based samples were captured to compare the results of the proposed setup against an established system. On every capturing device, the four inner-hand fingers (finger-IDs 2–5 and 7–10 according to ISO/IEC 19794-4 [30]) were captured. The capturing with the three capturing devices was conducted in two rounds. Figure 6 illustrates the capturing setups.

**Figure 7:**
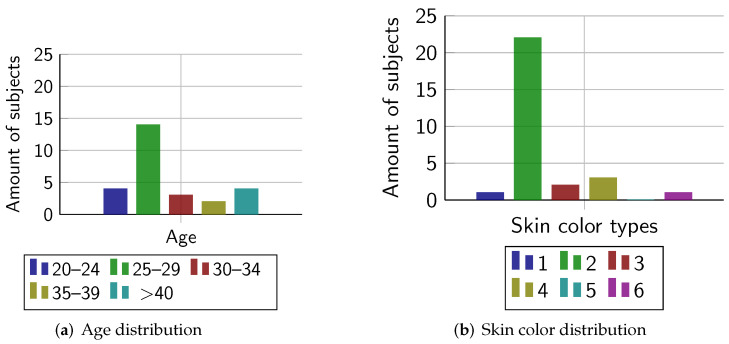
Distribution of age and skin color, according to Fitzpatrick metric [32] of the subjects.

To measure the biometric performance of the proposed system, we captured a database of 29 subjects. The age and skin color distribution can be seen in Figure 7. Table 3 summarizes the database-capturing method. During the capturing of one subject, failure-to-acquire (FTA) errors according to ISO/IEC 19795-1 [31] occurred on both contactless capturing devices. Interestingly, this was most likely caused by the length of the subject’s fingernails. For more information, the reader is referred to Section 7.6. In total, we captured and processed 1360 fingerprints.

### 4.2. Usability Study Design

A usability study was conducted with each subject after they had interacted with the capturing devices. Each subject was asked about their individual preferences in terms of hygiene and convenience during the capturing process. Parts of our usability study are based on [16,33]. We ensured that the questionnaire was as short and formulated as clearly as possible such that the participants understood all questions correctly [34].

The questionnaire is provided as supplemental material and it contains three parts. The first part contains questions about the subject’s personal preferences; questions 1.2b and 1.2c are aligned with Furman et al. [33]. Here, the different perceptions for personal hygiene before and during the COVID-19 pandemic were asked. The answer options of question 1.5 were rated by the capture subjects using the Rohrmann scale [35] (strongly disagree, fairly disagree, undecided, fairly agree, strongly agree). The questions were intended to find out the subjects’ perception regarding hygienic concerns during the fingerprint capturing process.

The second part of the questionnaire contains questions about the dedicated usability of a capturing device. The same questions were answered by the subject for both devices. This part was designed so that the same questions for both capturing devices were asked separately from each other in blocks. The intention behind this is to conduct comparisons between the different capturing devices. Again, the Rohrmann scale was used, and sub-questions were arranged randomly. In the last part of the questionnaire, the subjects were asked about their personal preference between both capturing devices. Here, the subjects had to choose one preferred capturing device.

## 5. Results

This section presents the biometric performance achieved by the entire recognition pipeline and the outcome of our usability study.

### 5.1. Biometric Performance

In our experiments, we first estimate the distributions of NFIQ2.0 scores for the captured dataset. Additionally, the biometric performance is evaluated employing open-source fingerprint recognition systems. The features (minutiae triplets—2D location and angle) are extracted using a neural network-based approach. In particular, the feature extraction method of Tang et al. [36] is employed. For this feature extractor, pretrained models are made available by the authors. To compare extracted templates, a minutiae pairing and scoring algorithm of the sourceAFIS system of Važan [37] is used^3^. We provide a script to set up the recognition pipeline along with our capturing and preprocessing pipeline.

**Figure 8:**
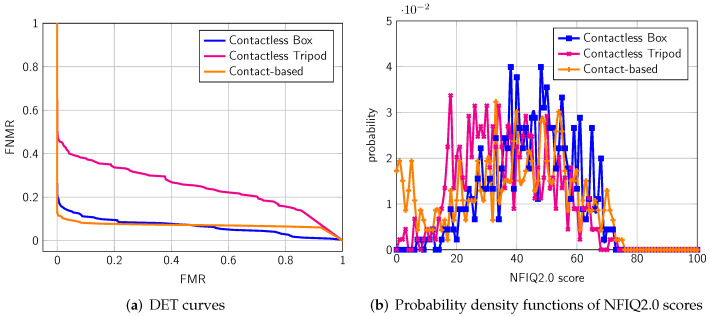
NFIQ2.0 score distribution and biometric performance obtained from single finger comparisons.

In the first experiment, we compare the biometric performance on all fingers between the different sub-datasets. From Table 4, we can see that the contactless box setup obtains an equal error rate (EER) of 10.71%, which is comparable to the contact-based setup (8.19%). Figure 8a presents the corresponding detection error trade-off (DET) curve, whereas Figure 8b shows the probability density functions of NFIQ2.0 scores. In contrast, the performance of the open setup massively drops to an EER of 30.41%. The corresponding NFIQ2.0 scores do not reflect this drop in terms of EER. Here, all three datasets have a comparable average score.

**Figure 9:**
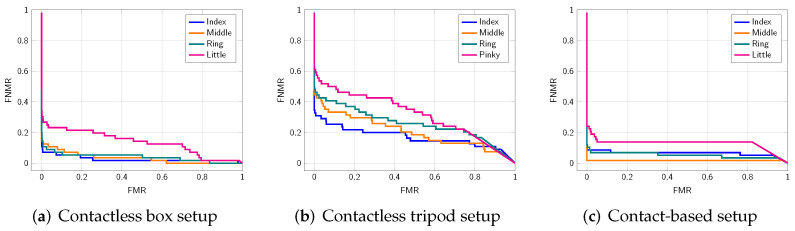
DET curves obtained from individual finger comparisons: index fingers (IDs 2, 7), middle fingers (IDs 3, 8), ring fingers (IDs 4, 9), and little fingers (IDs 5, 10).

**Figure 10:**
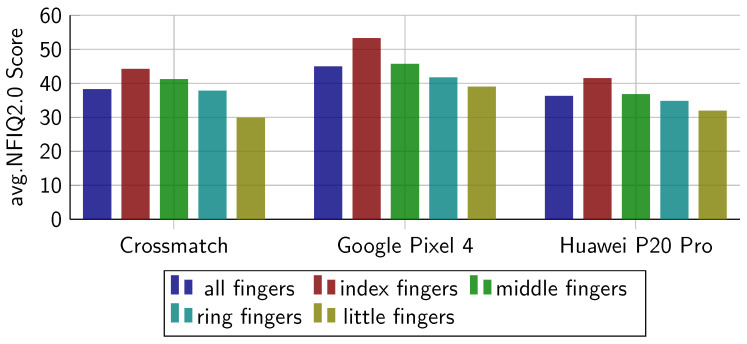
Averaged NFIQ2.0 scores obtained from the considered databases: average over all fingers (IDs 2–4, 6–10), index fingers (IDs 2, 7), middle fingers (IDs 3, 8), ring fingers (IDs 4, 9), and little fingers (IDs 5, 10).

In the second experiment, we compute the biometric performance for every finger separately^4^. From Table 5 and Figure 9, we can see that on all subsets, the performance of the little finger drops compared to the other fingers. On the contact-based sub-dataset, the middle finger has a much lower EER (1.72%) than the rest. This could be because it might be easiest for users to apply the correct pressure to the middle finger. From Figure 10, it is observable that there is only a small drop of NFIQ2.0 quality score on the little finger.

**Figure 11:**
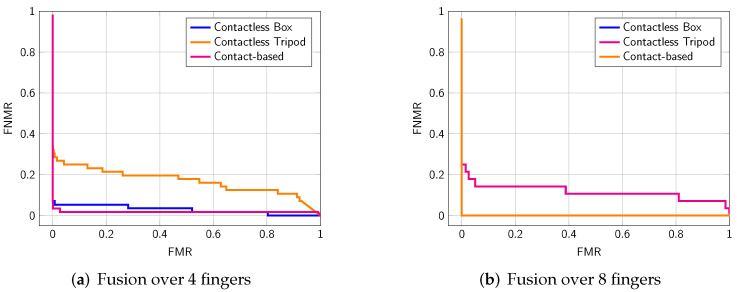
DET curves obtained in a fingerprint fusion approach: Fusion over the 4 inner-hand fingers of the left hand (IDs 2–4) and right hand (IDs 7–10) fusing (**a**) and fusion over 8 fingers of both inner hands (IDs: 2–4, 7–10) (**b**).

Further, we applied a score level fusion on four and eight fingers. Obtained EERs are summarized in Table 6. As expected, the fusion improves the EER on all sub-datasets. In particular, the fusion of eight fingers shows a huge performance gain (see Figure 11). The box setup and the contact-based sensor show an EER of 0%, which means that matches and nonmatches are completely separated. The open setup also achieves a considerably high performance gain through the fusion. Here, the inclusion of all fingers makes the process much more robust, especially in challenging environmental situations.

**Figure 12:**
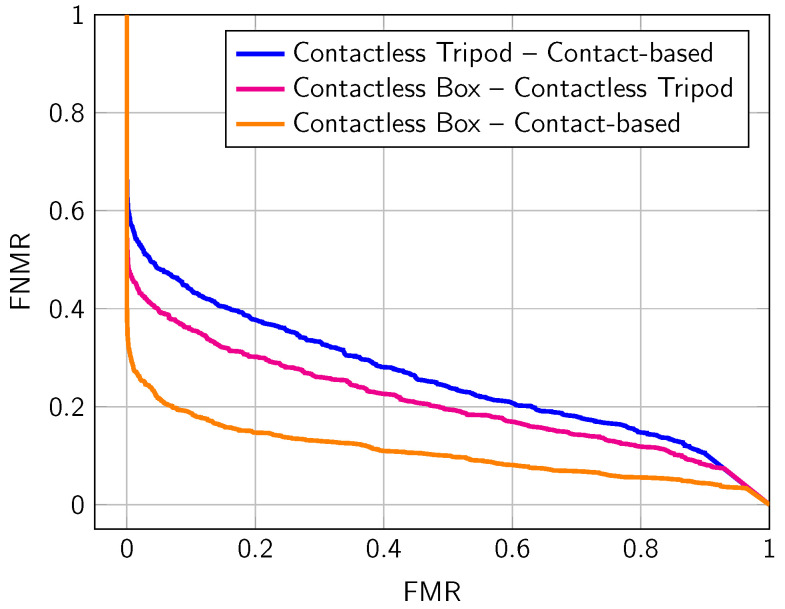
DET curves obtained from the interoperability of different subset of the collected data: Comparison of fingerprints captured with different setups. All captured fingers (finger-IDs 2–5 and 7–10) are considered.

In our last experiment, we analyze the interoperability between the different subsets of the collected data. Table 7 summarizes the EERs achieved by comparing the samples of different setups, and Figure 12 presents the corresponding DET curves. The contactless box setup shows a good interoperability to the contact-based setup (15.71%). The EER of the open setup again significantly drops (27.27% to contactless box and 32.02% to contact-based).

Table 8 compares the biometric performance and the average NFIQ2.0 scores of our proposed system to other publicly available databases. We used the algorithms from our method to process the contactless finger images to fingerprint samples.

We can see that the biometric performance on the fingerprint subcorpus of the MCYT bimodal database [38] and the Fingerprint Verification Contest 2006 (FVC06) [39] show a good performance. Moreover, the contactless subset of the Hong Kong Polytechnic University Contactless 2D to Contact-based 2D Fingerprint Images Database Version 1.0 (PolyU) [40] shows a competitive performance. Compared to these baselines, the performance achieved on our database is inferior, which is most likely due to the impact of the semisupervised acquisition scenario, as well as the use of hand disinfection measures during the COVID-19 pandemic. Section 6.1 further elaborates on these findings. It should also be noted that the PolyU Database was captured under very constrained environmental conditions with a single-finger capturing scenario and for a different purpose. For this reason, the obtained biometric performance cannot be directly compared to our method.

### 5.2. Usability Study

We present the results of our usability study based on the questionnaire introduced in Section 4.2. The questionnaire was answered by 27 subjects (8 female, 19 male). The subjects were between 22 and 60 years old (average age: 31.22 years, median age: 28 years). The age distribution is presented in Figure 7. The majority of subjects have used professional fingerprint scanners before this study. A large proportion of the 27 data subjects also use some type of fingerprint capturing device regularly (at least once per week), e.g., to unlock mobile devices.

**Figure 13:**
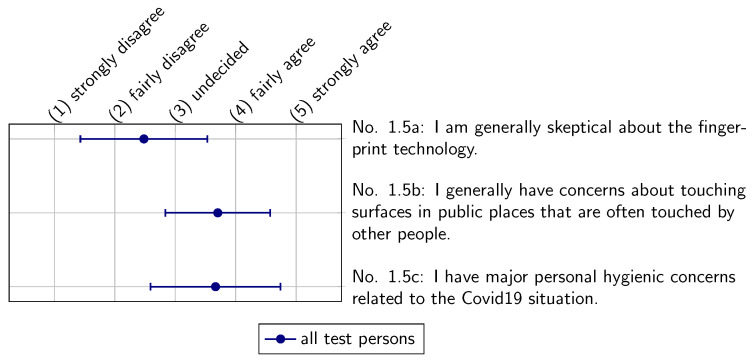
General assessment of fingerprint technology and hygienic concerns.

Figure 13 presents the perceptions of the subjects regarding general hygiene. The subjects in our study tend to have general concerns about touching surfaces in public places (Statement 1.5b). Moreover, the majority of the asked subjects have personal concerns related to the COVID-19 pandemic (Statement 1.5c). From the small difference in terms of perception before and during the COVID-19 pandemic, it could be inferred that the pandemic might have only a small influence on the general hygienic awareness of the subjects tested in our study.

**Figure 14:**
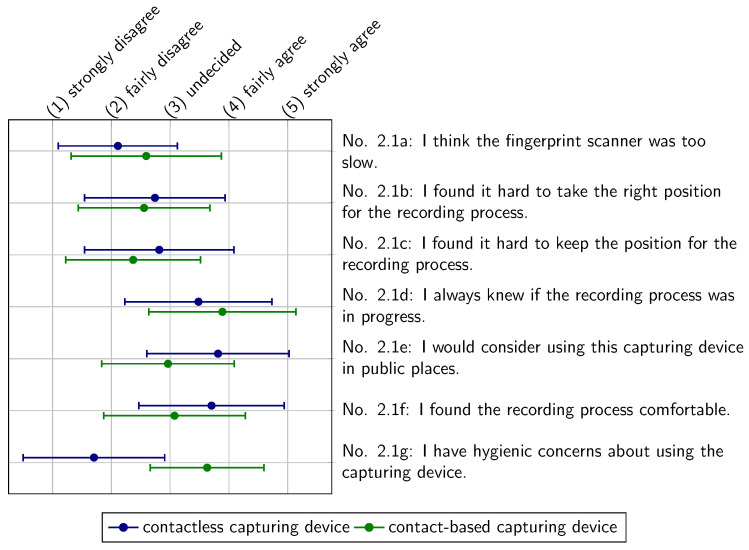
Usability assessment of the contactless and contact-based capturing device in comparison to each other.

The usability assessment of the contactless and contact-based capturing devices is presented in Figure 14. In most statements, both capturing devices were rated fairly similar by the asked subjects. The contactless capturing device has a slight advantage in terms of capturing speed (Statement 2.1a). The contact-based capturing device tends to be rated better in taking and keeping the capturing position during the whole process (Statements 2.1b and 2.1c). In addition, the subjects asked in our study found it slightly easier to assess whether the capturing process was running (Statement 2.1d). Moreover, it can be observed that the tested group prefer the comfort of the contactless device (Statement 2.1f). Most notably, the asked subjects might have less hygienic concerns using the contactless device in public places (Statements 2.1e and 2.1g). In these cases, a U-Test [41] shows a two-sided significance with a level of α=5%.

**Figure 15:**
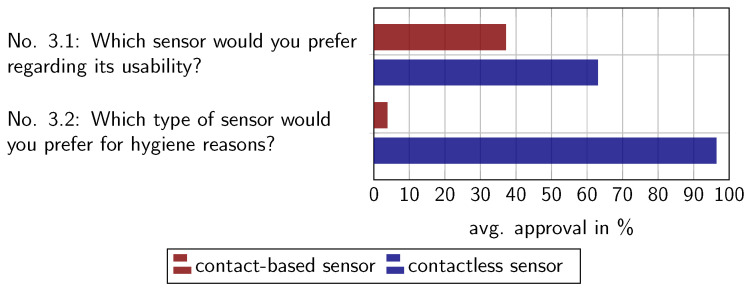
Comparative assessment of the capturing device type preference.

Figure 15 illustrates the comparative results. In a direct comparison of the different capturing device types, the advantage of hygiene might outweigh the disadvantages of hand positioning. The slight majority of subjects in our study might prefer the contactless capturing device over a contact-based one in terms of general usability (Question 3.1). Considering hygienic aspects, the majority of the asked subjects would choose the contactless capturing device over the contact-based one (Question 3.2). This correlates to the assessment of hygienic concerns of Statement 1.5c.

It should be noted that this study includes only a number of 27 subjects which might not be statistically sufficient to conduct a trustable census. In addition, as Figure 7 indicates, the age and skin color of the subjects are not distributed equally. For this reason, the results might not represent the general perception in society and should be treated with care.

## 6. Impact of the COVID-19 Pandemic on Fingerprint Recognition

The accuracy of some biometric characteristics may be negatively impacted by the COVID-19 pandemic. The pandemic and its related measures have no direct impact on the operation of fingerprint recognition. Nevertheless, there are important factors that may indirectly reduce the recognition performance and user acceptance of fingerprint recognition.

### 6.1. Impact of Hand Disinfection on Biometric Performance

The biometric performance drops due to dry and worn-out fingertips. Olsen et al. [42] showed that the level of moisture has a significant impact on the biometric performance of contact-based fingerprint recognition systems. The authors tested five capturing devices with normal, wet, and dry fingers. Dry fingers have especially been shown to be challenging. In addition, medical studies have shown that frequent hand disinfection causes dermatological problems [43,44]. The disinfection liquids dry out the skin and cause chaps in the epidermis and dermis.

**Figure 16:**
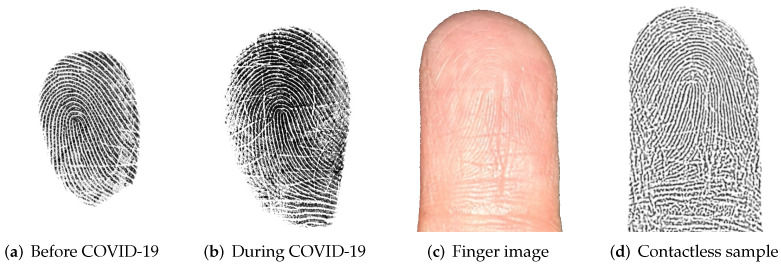
Four samples of the same subject: Sample (**a**) was captured before the COVID-19 pandemic using a contact-based capturing device, whereas samples (**b**–**d**) were captured during the COVID-19 pandemic. Samples (**a**,**b**) were captured with the same capturing device, whereas (**c**,**d**) are captured and processed using our method.

Thus, we can infer that regular hand disinfection leads to two interconnected problems which reduce the recognition performance: Dry fingers show low contrast during the capturing due to insufficient moisture. In addition, disinfection liquids lead to chaps on the finger surface. Figure 16 shows contact-based fingerprints captured before the COVID-19 pandemic (a) and during the COVID-19 pandemic (b). Both samples were captured from the same subject using the same capturing device. It is observable that sample (b) exhibits more impairments in the ridge-line pattern compared to sample (a). Moreover, the finger image (c) clearly shows chaps in the finger surface which are likely caused by hygienic measures. The processed contactless sample (d) shows these impairments, too.

The biometric performances reported for different databases presented in Table 8 also support these observations. Compared to the baseline of databases acquired before the COVID-19 pandemic, the performance achieved on our database is inferior. This is most likely caused by the impact of our semisupervised acquisition scenario, as well as the use of hand disinfection measures.

### 6.2. User Acceptance

Viruses, e.g., SARS-CoV-2, have four main transmission routes: droplet, airborne, direct contact, and indirect contact via surfaces. In the last case, an infected individual contaminates a surface by touching it. A susceptible individual who touches the surface afterwards has a high risk of infection via this indirect transmission route. Otter et al. [45] present an overview of the transmission of different viruses (including SARS coronaviruses) via dry surfaces. The authors conclude that SARS coronaviruses can survive for extended periods on surfaces and, for this reason, form a high risk of infection.

In large-scale implementations e.g., the Schengen Entry/Exit System (EES) [46] where many individuals contact the surface of a capturing device, the users are especially exposed to a major risk of infection. The only way to implement a safe contact-based fingerprint recognition in such application scenarios is to apply a disinfection of the capturing device after every subject.

Nevertheless, the requirement of touching a surface can lower the user’s acceptance of contact-based fingerprint recognition. The results of our usability studies in Section 5.2 show that the asked individuals are fairly skeptical about touching capturing device surfaces in public places and that (in a direct comparison) they would prefer a contactless capturing device. For this reason, the contactless capturing schemes could lead to a higher user acceptance. However, it should be noted that our tested group was very small and user acceptance is dependent on the capturing device design.

## 7. Implementation Aspects

This section summarizes aspects which are considered beneficial for practical implementation.

### 7.1. Four-Finger Capturing

As has been shown in previous works, our proposed recognition pipeline demonstrates that it is possible to process four fingerprints from a continuous stream of input images. This requires a more elaborated processing but has two major advantages:

- Faster and more accurate recognition process: Due to a larger proportion of finger area in the image, focusing algorithms work more precisely. This results in less misfocusing and segmentation issues.
- Improved biometric performance: The direct capturing of four fingerprints in one single capturing attempt is highly suitable for biometric fusion. As shown in Table 6, this lowers the EER without any additional capturing and with very little additional processing.

However, a major obstacle for contactless schemes is to capture the thumbs accurately and conveniently. In most environments, the best results are achieved with the inner-hand fingers facing upwards. This is ergonomically hard to achieve with thumbs.

### 7.2. Automatic Capturing and On-Device Processing

State-of-the-art smartphones feature powerful processing units which are capable to execute the described processing pipeline in a reasonable amount of time. We have shown that a robust and convenient capturing relies on automatic capturing with integrated plausibility checks. In addition, the amount of data which has to be transferred to a remote recognition workflow is reduced by on-device processing, and the recognition workflow can be based on standard components.

In a biometric authentication scenario, it can be especially beneficial to integrate the feature extraction and comparison into the mobile device. In this case, an authentication of a previously enrolled subject can be implemented on a standalone device.

### 7.3. Environmental Influences

**Figure 17:**
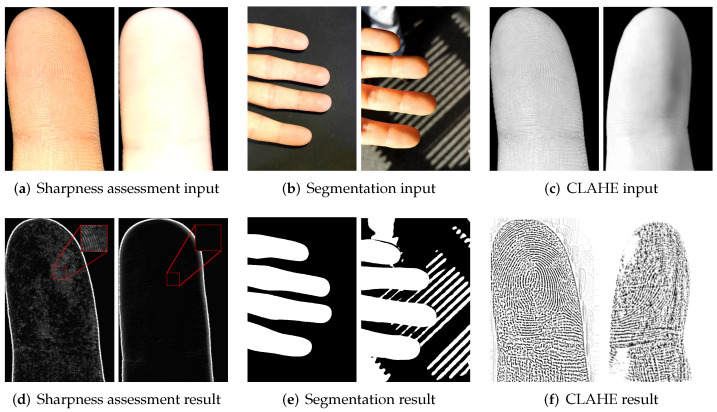
Illustration of accurate and challenging input images and corresponding result images for sharpness assessment (**a**,**d**), segmentation (**b**,**e**), and contrast adjustment (**c**,**f**). The left images of each block represent an accurate image; the right one—a challenging one.

Contactless fingerprint recognition in unconstrained environmental situations may be negatively affected by varying and heterogeneous influences. In our experiments, we showed that our contactless setup performs rather well under a semicontrolled environment. The performance of the same recognition pipeline drastically drops in an uncontrolled environment. Here, it is observable that different stages of the processing pipeline suffer from challenging environments:

- Focusing of the hand area needs to be very accurate and fast in order to provide sharp finger images. Here, a focus point which is missed by a few millimeters causes a blurred and unusable image. Figure 17a,d illustrate the difference between a sharp finger image and a slightly unfocused image with the help of a Sobel filter. Additionally, the focus has to follow the hand movement in order to achieve a continuous stream of sharp images. The focus of our tested devices tend to fail under challenging illuminations which was not the case in the constrained environment.
- Segmentation, rotation, and finger separation rely on a binary mask in which the hand area is clearly separated from the background. Figure 17b,e show examples of a successful and unsuccessful segmentation. Impurities in the segmentation mask lead to connected areas between the fingertips and artifacts at the border region of the image. This causes inaccurate detection and separation of the fingertips and incorrect rotation results. Because of heterogeneous background, this is more often the case in unconstrained setups.
- Finger image enhancement using the CLAHE algorithm normalizes dark and bright areas on the finger image. From Figure 17c,e, we can see that this also works on samples of high contrast. Nevertheless, the results of challenging images may become more blurry.

The discussed challenges lead to a longer capturing time and, for this reason, they lower the usability and user acceptance. Furthermore, the recognition performance in unconstrained environments is limited. Here, a weighing between usability and performance should be performed based on the intended use case of the capturing device. The quality assessments implemented in our scheme detect these circumstances and discard finger images with said shortcomings. More elaborated methods could directly adapt to challenging images, e.g., by changing the focusing method or segmentation scheme. This approach could lead to more robustness and hence improved usability in different environments.

### 7.4. Feature Extraction Strategies

**Figure 18:**
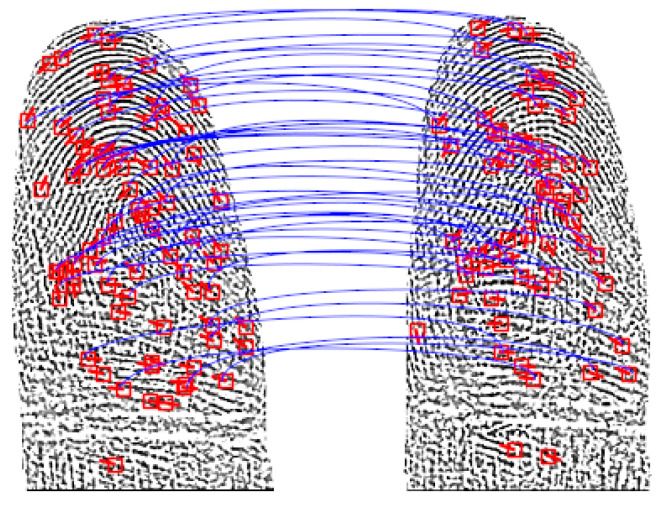
Illustration of a minutiae-based comparison of two contactless fingerprint samples. The features are extracted using the method described in Section 4. The blue lines indicate mated minutiae.

Feature extraction techniques are vital to achieve a high biometric performance. In our experiments, we used an open-source feature extractor which is able to process contact-based and contactless samples. Figure 18 shows an example of this minutiae-based feature extraction and comparison scheme. With this method, we were able to test the interoperability between capturing device types. Nevertheless, the overall performance may be improved by more sophisticated methods, e.g., commercial off-the-shelf-systems such as the VeriFinger SDK [47].

Dedicated contactless feature-extraction methods can increase the performance, as shown in [13,48]. Here, the authors were able to tune their feature extractor to their capturing and processing and they proposed an end-to-end recognition system.

Contactless fingerprint images do not correspond to the standardized 500 dpi resolution of contact-based capturing devices because of a varying distance between the capturing device and the fingertip. This challenge can be addressed in different ways:

Feature extractors and comparison algorithms which are robust against resolution differences provide an efficient capturing process. Here, metric scaling approaches or deep learning methods could be beneficial implementation strategies. A normalization to a predefined width of the fingerprint image such as proposed in this work (c.f. Figure 3) is also considered as beneficial, especially if off-the-shelf comparison algorithms are used. Countermeasures could also be implemented in the capturing stage. A fixed focal length calibrated on a suitable sensor-to-finger distance could reduce the variance in terms of size. This method could also be combined with an on-screen finger guidance, such as, e.g., that proposed by Carney et al. [14]. It should be noted that these approaches might be inferior for the system’s usability.

### 7.5. Visual Instruction

According to the presented results in Section 5.2, the visual feedback of the contactless capturing device has also been rated to be inferior compared to the contact-based one. Here, the smartphone display is well suited to show further information about the capturing process. Additionally, an actionable feedback can be given on the positioning of the fingers, as suggested in [14].

### 7.6. Robust Capturing of Different Skin Colors and Finger Characteristics

An important implementation aspect of biometric systems is that they must not discriminate certain user groups based on skin color or other characteristics. During our database-capturing, subjects of different skin color types were successfully captured. Nevertheless, it must be noted that the amount of subjects is too small to make a general statement about the fairness of the presented approach.

As already mentioned in Section 4.1, we observed one single failure-to-acquire (FTA) during our database acquisition. Most likely the cause for this was that the subject had very long fingernails which were segmented as finger area. Here, the plausibility check during the segmentation failed and a capturing of the subject was not possible. To overcome this flaw, a fingernail detection could be implemented in the segmentation workflow.

## 8. Conclusions

In this work, we proposed a fingerprint recognition workflow for state-of-the-art smartphones. The method is able to automatically capture the four inner-hand fingers of a subject and process them to separated fingerprint images. With this scheme, we captured a database of 1360 fingerprints from 29 subjects. Here, we used two different setups: a box setup with constrained environmental influences, and a tripod setup. Additionally, we captured contact-based fingerprints as baseline. During a usability study, after the capturing, the subjects were asked about their experience with the different capturing device types.

Our investigations show that the overall biometric performance of the contactless box setup is comparable to the contact-based baseline, whereas the unconstrained contactless tripod setup shows inferior results. All setups benefit from a biometric fusion. A further experiment on the interoperability between contactless and contact-based samples (box setup) shows that the performance drops only slightly.

The presented usability study shows that the majority of users prefer a contactless recognition system over a contact-based one for hygienic reasons. In addition, the usability of the contactless capturing device was seen as slightly better. Nevertheless, the user experience of the tested contactless devices can be further improved.

The COVID-19 pandemic also has an influence on the performance and acceptance of fingerprint recognition systems. Here, hygienic measures lower the recognition performance and users show more concern regarding touching surfaces in public areas.

Our proposed method forms a baseline for a mobile automatic contactless fingerprint recognition system and is made publicly available. Researchers are encouraged to integrate their algorithms into our system and contribute to a more accurate, robust, and secure contactless fingerprint recognition scheme.

## Figures and Tables

**Table 2:** Technical specifications of the contactless capturing devices used during the data acquisition.

Device	Google Pixel 4	Huawei P20 Pro
Chipset	Snapdragon 855	Kirin 970
CPU	Octa-core	
Ram	6 GB	
Camera	12.2 MP, f/1.7, 27 mm	40 MP, f/1.8, 27 mm
Flash mode	Always on	
Avg. system load	∼84%	∼73%

**Table 3:** Overview of selected recognition workflows with biometric performance.

Type	Setup	Device	Subjects Captured	Rounds	Samples
Contactless	box	Google Pixel 4	28	2	448
Contactless	tripod	Huawei P20 Pro	28	2	448
Contact-based	-	Crossmatch Guardian 100	29	2	464

**Table 4:** Overview of the NFIQ2.0 quality scores and the EER of all captured fingers (finger-IDs 2–5 and 7–10) separated by sensors.

Capturing Device	Subset	Avg. NFIQ2.0 Score	EER (%)
Contactless box	All fingers	44.80 (±13.51)	10.71
Contactless tripod	All fingers	36.15 (±14.45)	30.41
Contact-based	All fingers	38.15 (±19.33 )	8.19

**Table 5:** Overview of the NFIQ2.0 quality scores and the EER of individual fingers: index fingers (IDs 2, 7), middle fingers (IDs 3, 8), ring fingers (IDs 4, 9), and little fingers (IDs 5, 10).

Capturing Device	Fingers	Avg. NFIQ2.0 Score	EER (%)
Contactless box	Index fingers	53.16 (±11.27)	7.14
Contactless box	Middle fingers	45.59 (±11.06)	8.91
Contactless box	Ring fingers	41.57 (±12.89)	7.14
Contactless box	Little fingers	38.88 (±14.21)	21.43
Contactless tripod	Index fingers	41.38 (±14.29)	21.81
Contactless tripod	Middle fingers	36.68 (±13.01)	28.58
Contactless tripod	Ring fingers	34.68 (±14.28)	29.62
Contactless tripod	Little fingers	31.79 (±14.63)	38.98
Contact-based	Index fingers	44.06 (±17.53 )	8.62
Contact-based	Middle fingers	41.08 (±19.71 )	1.72
Contact-based	Ring fingers	37.68 (±17.08 )	6.90
Contact-based	Little fingers	29.78 (±19.94 )	13.79

**Table 6:** Overview of the EER in a fingerprint fusion approach: Fusion over the 4 inner-hand fingers of the left hand (IDs 2–4) and right hand (IDs 7–10) fusing and fusion over 8 fingers of both inner hands (IDs: 2–4, 7–10).

Capturing Device	Fusion Approach	EER (%)
Contactless box	4 fingers	5.36
Contactless box	8 fingers	0.00
Contactless tripod	4 fingers	21.42
Contactless tripod	8 fingers	14.29
Contact-based	4 finger	2.22
Contact-based	8 finger	0.00

**Table 7:** Overview of the interoperability of different subset of the collected data: Comparison of fingerprints captured with different setups. All captured fingers (finger-IDs 2–5 and 7–10) are considered.

Capturing Device A	Capturing Device B	EER (%)
Contactless box	Contactless tripod	27.27
Contactless box	Contact-based	15.71
Contactless tripod	Contact-based	32.02

**Table 8:** Average NFIQ2.0 scores and biometric performance obtained from contactless and contact-based databases including the fingerprint subcorpus of the MCYT Database [38], the FVC2006 Database [39] and the Hong Kong Polytechnic University Contactless 2D to Contact-based 2D Fingerprint Images Database Version 1.0 [40].

Database	Subset	Avg. NFIQ2.0 Score	EER (%)
MCYT	dp	37.58 (±15.17)	0.48
	pb	33.02 (±13.99)	1.35
FVC06	DB2-A	36.07 (±9.07)	0.15
PolyU	Contactless session 1	47.71 (±10.86)	3.91
	Contactless session 2	47.08 (±13.21)	3.17
Our database	Contact-based	38.15 (±19.33 )	8.19
	Contactless box	44.80 (±13.51)	10.71

## Data Availability

Not applicable.

## Notes

1 Source code will be made available at https://gitlab.com/jannispriesnitz/mtfr (accessed on 8 December 2021).

2 Due to privacy regulations, it is not possible to make the database collected in this work publicly available.

3 The original algorithm uses minutiae quadruplets, i.e., additionally considers the minutiae type (e.g., ridge ending or bifurcation). As only minutiae triplets are extracted by the used minutiae extractors, the algorithm was modified to ignore the type information.

4 In this experiment, we consider only the same finger-IDs from a different subject as false match.
